# Glyoxalase 1 expression is associated with an unfavorable prognosis of oropharyngeal squamous cell carcinoma

**DOI:** 10.1186/s12885-017-3367-5

**Published:** 2017-05-26

**Authors:** Nele Kreycy, Christiane Gotzian, Thomas Fleming, Christa Flechtenmacher, Niels Grabe, Peter Plinkert, Jochen Hess, Karim Zaoui

**Affiliations:** 1 Department of Otolaryngology, Head and Neck Surgery, University Hospital Heidelberg, Im Neuenheimer Feld 400, D-69120 Heidelberg, Germany; 2 Department of Medicine I and Clinical Chemistry, University Hospital Heidelberg, Heidelberg, Germany; 3 Institute of Pathology, University Hospital Heidelberg, Heidelberg, Germany; 4 Medical Oncology, National Center for Tumor Diseases (NCT) and Hamamatsu Tissue Imaging and Analysis Center (TIGA), BIOQUANT, Heidelberg, Germany; 5 Department of Otolaryngology, Head and Neck Surgery, University Hospital Heidelberg and Research Group Molecular Mechanisms of Head and Neck Tumors, German Cancer Research Center (DKFZ), Heidelberg, Germany

**Keywords:** Argpyrimidine, Colony-forming assay, Disease-specific survival, Glyoxalase 1, Head and neck cancer, Methylglyoxal, Oropharyngeal squamous cell carcinoma, Reactive metabolites, Tissue microarray

## Abstract

**Background:**

Glyoxalase 1 is a key enzyme in the detoxification of reactive metabolites such as methylglyoxal and induced Glyoxalase 1 expression has been demonstrated for several human malignancies. However, the regulation and clinical relevance of Glyoxalase 1 in the context of head and neck squamous cell carcinoma has not been addressed so far.

**Methods:**

Argpyrimidine modification as a surrogate for methylglyoxal accumulation and Glyoxalase 1 expression in tumor cells was assessed by immunohistochemical staining of tissue microarrays with specimens from oropharyngeal squamous cell carcinoma patients (*n* = 154). Prognostic values of distinct Glyoxalase 1 staining patterns were demonstrated by Kaplan-Meier, univariate and multivariate Cox proportional hazard model analysis. The impact of exogenous methylglyoxal or a Glyoxalase 1 inhibitor on the viability of two established tumor cell lines was monitored by a colony-forming assay in vitro.

**Results:**

Glyoxalase 1 expression in tumor cells of oropharyngeal squamous cell carcinoma patients was positively correlated with the presence of Argpyrimidine modification and administration of exogenous methylglyoxal induced Glyoxalase 1 protein levels in FaDu and Cal27 cells in vitro. Cal27 cells with lower basal and methylglyoxal-induced Glyoxalase 1 expression were more sensitive to the cytotoxic effect at high methylgyoxal concentrations and both cell lines showed a decrease in colony formation with increasing amounts of a Glyoxalase 1 inhibitor. A high and nuclear Glyoxalase 1 staining was significantly correlated with shorter progression-free and disease-specific survival, and served as an independent risk factor for an unfavorable prognosis of oropharyngeal squamous cell carcinoma patients.

**Conclusions:**

Induced Glyoxalase 1 expression is a common feature in the pathogenesis of oropharyngeal squamous cell carcinoma and most likely represents an adaptive response to the accumulation of cytotoxic metabolites. Oropharyngeal squamous cell carcinoma patients with a high and nuclear Glyoxalase 1 staining pattern have a high risk for treatment failure, but might benefit from pharmacological targeting Glyoxalase 1 activity.

**Electronic supplementary material:**

The online version of this article (doi:10.1186/s12885-017-3367-5) contains supplementary material, which is available to authorized users.

## Background

The Warburg effect describes a condition in which cells with high proliferative activity rely on glycolysis as a major source of energy rather than oxidative phosphorylation [1]. High glycolytic activity is a characteristic feature of tumor cells in pre-malignant lesions as an adaptation to intermitted hypoxia and is particularly marked in invasive tumors [2]. As a consequence of accelerated glycolysis cancer cells accumulate endogenous dicarbonyl compounds such as methylglyoxal (MG), a highly reactive and potent glycating agent [3]. Insufficient metabolism of MG causes stable modifications of proteins, e.g. modification at arginine residues known as argpyrimidine (AP), nucleotides and lipids, leading to accelerated levels of advanced glycation end products (AGEs). These modifications cause serious damage to the functional integrity of the genome and the proteome [4]. The carbonyl stress related to MG has been primarily described in the pathology of diabetes, where accumulation of AGEs is a common event in the manifestation and maintenance of late complications [5, 6]. MG is a potent cytotoxic compound and its accumulation exerts anti-tumor activity suggesting its potential use as therapeutic agent in distinct cancers [7]. However, several studies also reported a putative pro-tumorigenic effect of MG mainly due to post-translational modification of cancer-related proteins, indicating an impact of the global cellular context.

In mammalian cells, MG is detoxified by the glyoxalase system, an ubiquitous cellular defense mechanism comprising glyoxalase 1 (GLO1), glyoxalase 2 (GLO2/HAGH) and a catalytic amount of reduced glutathione [3]. The glyoxalase system has been considered to maintain tumor cell activity and viability by preventing cellular suicide due to MG accumulation. Indeed, GLO1 gene amplification and elevated expression is a common feature in the progression of multiple human malignancies, including gastric cancer [8], colorectal cancer [9], breast cancer [10], liver cancer [11, 12], skin tumors [13, 14], and prostate cancer [15]. In some tumor entities GLO1 expression was associated with advanced tumor stages or drug resistance [3, 8, 10, 16]. The increase in GLO1 expression and activity most likely resembles a strategy adopted by aggressive cancer cells as a defense mechanism against glycation damage induced by high intracellular MG levels as a consequence of elevated glycolytic activity or under therapeutic conditions [3]. Thus, GLO1 plays a vital role in tumor initiation, malignant progression as well as treatment failure, and could serve as promising target for anti-cancer therapy.

So far, neither the expression of GLO1 nor its impact on malignant progression or prognosis have been addressed for head and neck squamous cell carcinoma (HNSCC). HNSCC is one of the most common human malignancies with an annual incidence of 600,000 new cases worldwide [17]. Traditional risk factors are tobacco and alcohol abuse and more recently, infection by high-risk types of human papilloma virus (HPV), especially HPV16, has been related to an escalating incidence of oropharyngeal squamous cell carcinoma (OPSCC) [18, 19]. Despite aggressive and multimodal therapy mainly consisting of surgery, radiotherapy and platinum-based chemotherapy, the survival of patients with advanced HNSCC has only marginally improved during the past decades. Consequently, appropriate treatment of HNSCC patients is still a major challenge and there is an urgent demand for new concepts of more effective and less toxic therapy.

## Methods

### Patient samples and tissue microarray

Tumor specimens for this retrospective study were obtained from patients with primary OPSCC, who have given informed consent to participate and were treated at the University Hospital Heidelberg between 1990 and 2008. Formaldehyde fixed and paraffin embedded tissue specimens were provided by the tissue bank of the National Center for Tumor Disease (Institute of Pathology, University Hospital Heidelberg) after approval by the local institutional review board (ethic vote: 176/2002 and 206/2005). The study was performed according to the ethical standards of the Declaration of Helsinki. For all tumor samples, clinical and follow-up data were available from the Department of Otolaryngology, Head and Neck Surgery at the University Hospital Heidelberg and are listed in Additional file 1: Table S1. HPV16 DNA and viral transcript status was determined previously [20] and generation of tissue microarrays has been described elsewhere [21].

### Immunohistochemical staining and scoring system

Tissue microarrays were assembled with independent cores from distinct areas of a formalin-fixed and paraffin embedded pre-treatment tumor tissue and for most tumors at least two independent probes were available. Tissue microarrays were incubated with anti-AP or anti-GLO1 antibodies that are listed in Additional file 2: Table S2. Immunostaining was visualized with the TSA Amplification Kit (Perkin Elmer, Rodgau, Germany) and DAB peroxidase substrate (Vector Laboratories, Burlingame, USA) according to the manufacturers instructions. Counterstaining was done by hematoxylin to visualize tissue integrity. Stained tissue microarrays were scanned using the Nanozoomer HT Scan System (Hamamatsu Photonics, Japan). Protein expression was evaluated by three independent observers using the NDP Viewer software (version 1.1.27). For AP the staining intensity (score 1 = no, score 2 = low, score 3 = moderate, score 4 = high) was evaluated. For GLO1 the relative amount of positive tumor cells (score 1 = no positive cell, score 2 ≤ 33%, 33% > score 3 ≤ 66%, score 4 > 66%) and the staining intensity (score 1 = no, score 2 = low, score 3 = moderate, score 4 = high) was evaluated (Additional file 3: Table S3). Both scores were significantly correlated (Pearson correlation coefficient 0.696, *p* < 0.005 and Spearman correlation coefficient 0.586, *p* < 0.005) and multiplied to calculate the final immunoreactivity score (IRS, range 1–16). The cut-off value for further analysis was: GLO1^high^ > 8 and GLO1^low^ ≤ 8. The intracellular staining of GLO1 was evaluated as predominant nuclear (GLO1^nuc^) versus cytoplasmic (GLO1^cyt^) (Additional file 4: Table S4).

### Cell culture experiments

Human cell lines (FaDu and Cal27) were purchased from ATCC (http://www.lgcstandards-atcc.org; catalog numbers: HTB-43™ and CRL-2095™) and were maintained in http://www.sigmaaldrich.com (Sigma, Germany) supplemented with 10% fetal bovine serum (Invitrogen, Germany), 2 mM L-Glutamine (Invitrogen, Germany) and 50 μg/ml Penicillin-Streptomycin (Invitrogen, Germany) in a humidified atmosphere of 6% CO2 at 37 °C. Authentication of both cell lines was confirmed by the Multiplex Human Cell Line Authentication Test (Multiplexion, Germany). For Western blot analysis 1 × 10^6^ FaDu or Cal27 cells were seeded on 96 mm TC plates and cultured over night. Cells were treated with indicated concentrations of MG and 24 h following administration cells were harvested for protein lysates. MG was synthesized by acid hydrolysis as described [22], and the concentration of the stock solution was determined by derivatization with aminoguanidine [23]. The cell permeable GLO1 inhibitor, S-*p*-bromobenzylglutathione cyclopentyl diester (BrBzGSHCp_2_) was prepared and characterized as described [24, 25]. For colony formation assays 100 and 300 FaDu cells or 300 and 1000 Cal27 cells were seeded per 6-well plate, respectively. Cells were treated with the indicated concentration of MG or the GLO1 inhibitor every second day. 10 days upon seeding cells were PFA-fixed and the amount of colonies was determined after crystal violet staining as described in [26]. The survival fraction was computed according to [27].

### Protein isolation and Western blot analysis

Whole cell protein lysate was extracted using RIPA (Radioimmunoprecipitation assay) buffer [28] and protease and phosphatase inhibitor cocktails (Sigma-Aldrich). 20 μg of denatured protein were separated by Sodiumdodecylsulfate-polyacrylamide gel electrophoresis (SDS-PAGE) and transferred to polyvinyl difluoride (PVDF) membranes (Millipore, Germany). After blocking with 5% milk (Roth, Germany), membranes were incubated with primary and horseradish peroxidase coupled-secondary antibodies, which are listed in Supplementary Table S2. Membranes were incubated in enhanced chemiluminiscence solution (Thermo Scientific, Germany) and developed with an E.O.S. developer (Agfa, Germany).

### Quantification of MG levels by HPLC

The concentrations of MG in supernatant of cultured cells were determined by derivatization with 1,2-diamino-4,5-dimethoxybenzene and HPLC of the quinoxaline adduct by fluorescence detection as described elsewhere [22, 29].

### Statistical analysis

Statistical analysis was conducted with the IBM SPSS Statistics software (version 21). Differences between subgroups were assessed using Chi square or Fisher’s exact tests. The method of Kaplan–Meier was used to estimate survival distributions and differences between groups were determined by log-rank tests. Univariate and multivariate Cox proportional hazard model was used to assess the association between patient subgroups and progression-free or disease-specific survival. In all statistical tests, a *p*-value of 0.05 or below was considered as statistically significant.

## Results

### Detection of AP modification and correlation with GLO1 expression in primary oropharyngeal squamous cell carcinoma

**Fig. 1:**
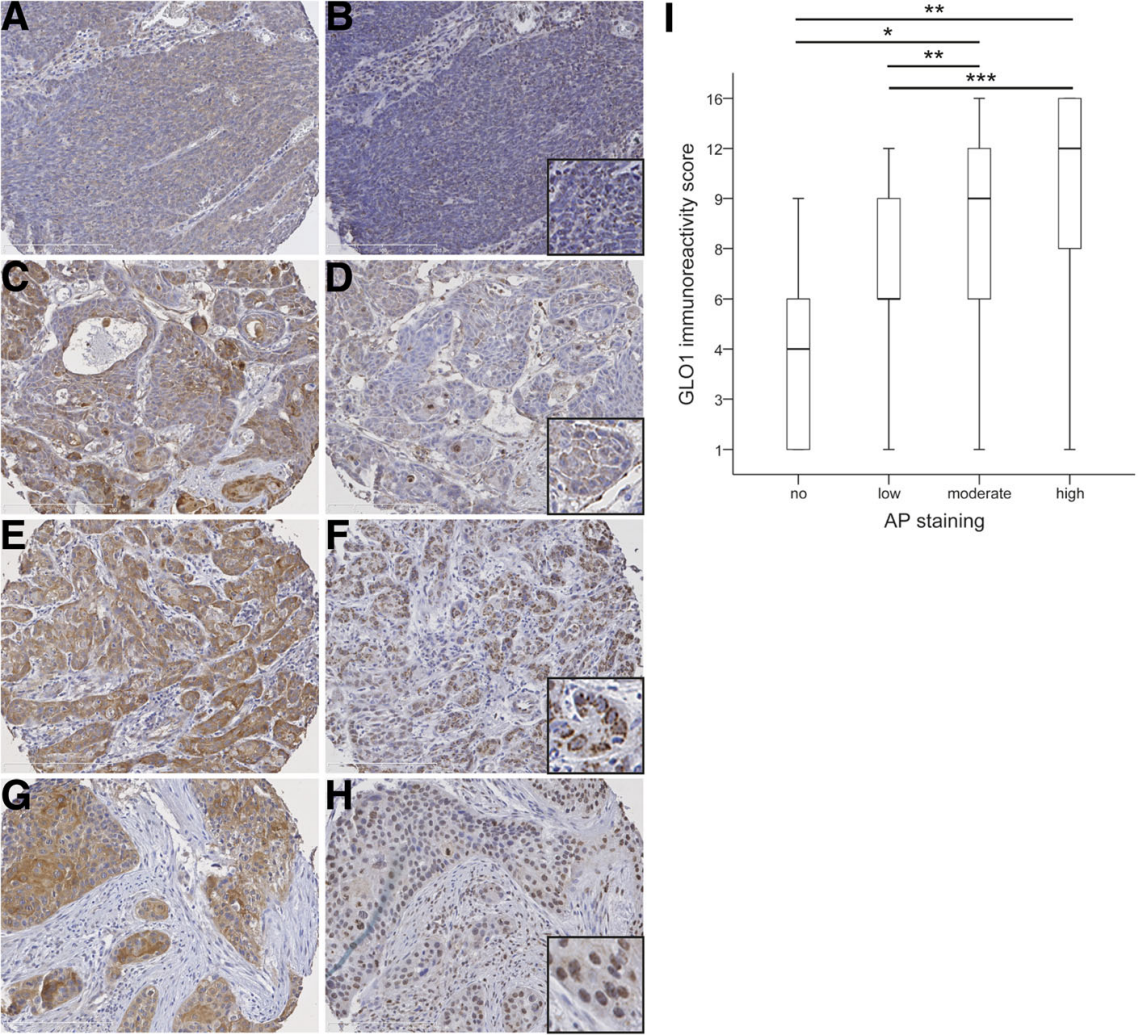
Detection of AP modifications and GLO1 protein expression in tumor cells of OPSCC. Representative pictures of an immunohistochemical staining with an anti-AP antibody (brown staining) demonstrate tumor sections with low (**a**), moderate (**c**) or high staining (**e** and **g**). Serial sections were analyzed with an anti-GLO1 antibody and revealed a heterogeneous staining pattern (brown signal) with low (**b**), moderate (**d**) and high immunoreactivity scores (**f** and **h**), considering staining intensity and relative amount of positive tumor cells. GLO1 staining was detected either in the cytoplasm (**f**) or the nucleus (**h**) of tumor cells. **i** Boxplot displays the median and 25% to 75% percentile of the GLO1 immunoreactivity score in subgroups of OPSCC patients (*n* = 134) with no, low, moderate or high AP staining. *p ≤ 0.05, **p ≤ 0.005 and ***p ≤ 0.0005. Counterstaining was done with hematoxylin to visualize tissue architecture; white bar indicates 200 μm

Tissue microarrays were analyzed by immunohistochemistry with an anti-AP antibody as a surrogate marker for MG accumulation in tumor cells of primary OPSCC. In parallel, GLO1 protein levels were analyzed by immunohistochemistry on serial sections of tissue microarrays. Data on AP and GLO1 immunoreactivity were available for tumor specimens of 134 patients, and revealed no AP staining in 3.7% (*n* = 5), low staining pattern in 20.9% (*n* = 28), moderate staining pattern in 31.4% (*n* = 42), and a high staining pattern in 44% (*n* = 59) (Fig. 1A, C, E and G). A heterogeneous staining pattern was also evident for GLO1 considering the staining intensity as well as the relative amount of positive tumor cells (Fig. 1B, D, F and H). It is worth noting that in addition to the expected positive staining in the cytoplasm of tumor cells (Fig. 1F), we also detected a prominent nuclear GLO1 staining in a substantial amount of OPSCC samples (Fig. 1H). Comparison of the relative GLO1 immunoreactivity score in subgroups of OPSCC patients with no, low, moderate or high AP staining demonstrated a significant positive correlation (Fig. 1I).

### Impact of MG on GLO1 expression and viability of tumor cell lines

**Fig. 2:**
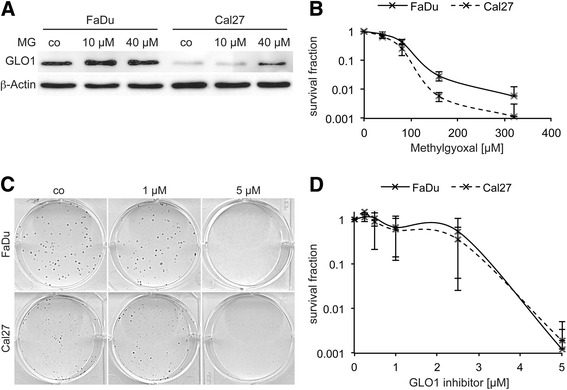
Impact of MG and a GLO1 inhibitor on colony formation of tumor cells. Western blot analysis with whole cell lysate demonstrates higher basal GLO1 expression in control treated (co) FaDu as compared to Cal27 cells and MG-induced up-regulation of GLO1 protein levels in both cell lines (**a**). Detection of β-Actin served as control for quantity and quality of protein lysates. **b** The cytotoxic effect of MG on FaDu (black line) and Cal27 cells (dashed line) was assessed by a colony-forming assay and the graph indicates the mean value ±SD of the survival fraction at the indicated MG concentration of three independent experiments. The impact of GLO1 inhibition (1 and 5 μM S-*p*-bromobenzylglutathione cyclopentyl diester) on the viability of FaDu and Cal27 cells was assessed by a colony-forming assay (**b**) and the graph in (**c**) displays the mean value ±SD of the survival fraction at the indicated concentration of the GLO1 inhibitor from three independent experiments

The data presented so far suggested that induced GLO1 expression is a compensatory mechanism of OPSCC tumor cells to counteract and survive the accumulation of MG. To further support this assumption FaDu and Cal27, two well-established head and neck cancer cell lines, were cultured in the presence of increasing amounts of MG. In both cell lines, administration of exogenous MG increased GLO1 protein levels as determined by Western blot analysis (Fig. 2A). It is worth noting that GLO1 levels were lower in Cal27 as compared to FaDu cells under control conditions as well as the presence of exogenous MG.

Next, the cytotoxic profile of MG treatment on tumor cell viability was analyzed by a colony-forming assay. No major difference between both cell lines was found at lower MG concentrations (≤ 40 μM, Additional file 5: Fig. S1). However, at higher MG concentrations FaDu cells displayed an improved survival as compared to Cal27 cells, which was statistical significant at a MG concentration of 160 μM (*p* ≤ 0.049, Fig. 2B). These data indicated a better tolerance of tumor cells with higher basal or inducible GLO1 expression under conditions of MG accumulation.

### Impact of GLO1 inhibition on tumor cell survival

To assess the consequence of GLO1 inhibition on tumor cell survival we cultured both cell lines in the presence of the cell permeable inhibitor S-*p*-bromobenzylglutathione cyclopentyl diester [3]. Quantification of MG levels in cell culture supernatant after treatment with the GLO1 inhibitor revealed a concentration dependent increase in FaDu but not Cal27 cells (Additional 6: Fig. S2). However, both cell lines showed a comparable decrease in the survival fraction with increasing amount of the GLO1 inhibitor (Fig. 2C-D). These data strongly suggested that the cytotoxic effect of GLO1 inhibition does not exclusively rely on an accumulation of MG levels.

### Prognostic value of the GLO1 staining pattern

**Fig. 3:**
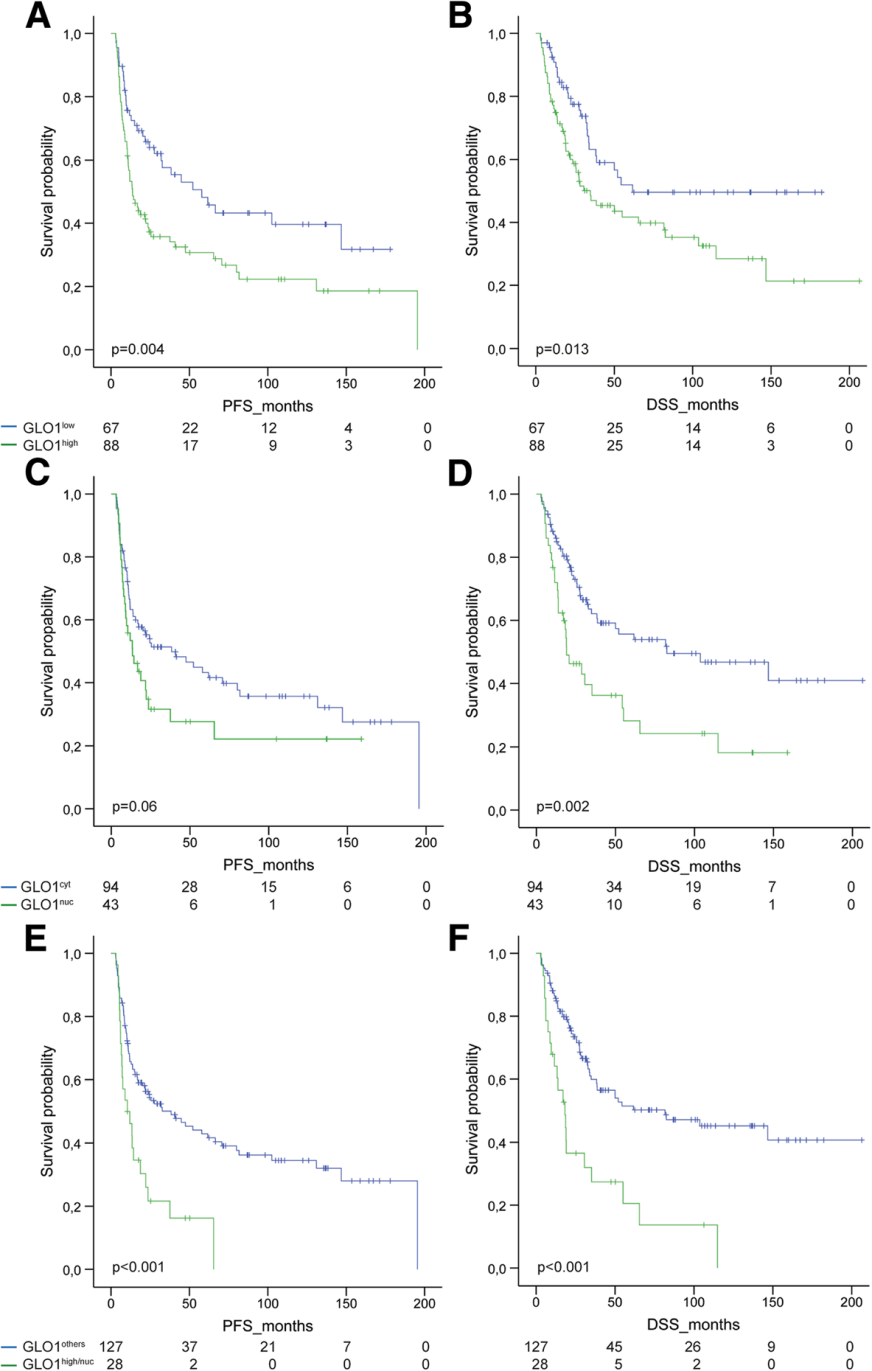
High expression and nuclear localization of GLO1 correlates with unfavorable survival. The prognostic value of high versus low GLO1 expression (**a-b**) and its predominant cytoplasmic versus nuclear localization (**c-d**) was assessed for progression-free (PFS, left panel) and disease-specific survival (DSS, right panel) in a Kaplan-Meier plot. The worst outcome was observed for OPSCC patients with a combined high and nuclear GLO1 staining as compared to all other staining patterns (**e-f**). *P* values were calculated by a log-rank test

To evaluate the clinical relevance of GLO1 staining patterns in the cohort of OPSCC patients, subgroups with a low (GLO1^low^, *n* = 67) or a high immunoreactivity score (GLO1^high^, *n* = 89) were identified. Furthermore, we considered a cytoplasmic (GLO1^cyt^, *n* = 94) or a predominant nuclear staining (GLO1^nuc^, *n* = 43) in the subgroup of OPSCC patients with a positive GLO1 staining. A GLO1^high^ staining was significantly correlated with shorter progression-free (PFS) and disease-specific survival (DSS), while nuclear GLO1 staining revealed a trend towards shorter PFS and was significantly correlated with shorter DSS (Fig. 3A-D). These findings strongly indicated that a nuclear GLO1 accumulation in tumor cells with a high expression serves as a risk factor for unfavorable prognosis of OPSCC patients. This assumption was further assessed by a combinatorial subgroup analysis taking into account both features (GLO1^high/nuc^) in comparison to all other staining patterns (GLO1^others^), and confirmed a highly significant shorter PFS and DSS (Fig. 3E-F). Moreover, a high and nuclear GLO1 staining was significantly associated with a larger tumor size (Table 1), but not with any other patient characteristic tested (e.g. gender, age, TNM status, pathological grading, clinical staging, alcohol and tobacco consumption or HPV status). Next, multivariate Cox proportional hazard model analysis confirmed that a GLO1^high/nuc^ staining pattern served as independent risk factors for unfavorable PFS and DSS (Table 2), taking into consideration relevant prognostic risk factors based on univariate analysis (Additional 7: Table S5).

**Table 1:** Correlation between GLO1^high/nuc^ staining and patient characteristics

		GLO1^high/nuc^		GLO1^others^		
Features	Category	N	%	N	%	*p* value^3^
Age [years]	< 58.25	18	64.3	63	49.2	0.148
	≥ 58.25	10	35.7	65	50.8	
Gender	Male	20	71.4	96	75.0	0.695
	Female	8	28.6	32	25.0	
T status	T1-T2	6	21.4	64	50.4	**0.005**
	T3-T4	22	78.6	63	49.6	
N status	N0	6	21.4	25	19.7	0.835
	N+	22	78.6	102	80.3	
M status	M0	25	92.6	120	96.0	0.444
	M+	2	7.4	5	4.0	
Pathological grading	G1–2	14	66.7	62	56.4	0.381
	G3	7	33.3	48	43.6	
Clinical staging	I-III	5	17.9	44	34.6	0.084
	IV	23	82.1	83	65.4	
Alcohol	no/former	3	11.1	29	24.2	0.137
	current	24	88.9	91	75.8	
Tobacco	no/former	3	11.1	29	23.8	0.147
	current	24	88.9	93	76.2	
HPV	non-related^1^	24	88.9	83	75.5	0.130
	related^2^	3	11.1	27	24.5	

^*1*^ *viral DNA-negative or DNA-positive but transcript-negative;* ^*2*^ *viral DNA- and transcript-positive according to* [20]*;* ^*3*^ *Chi-square test*

Significant results (*p* <0.05) (including the HR, 95% CI) are marked in boldface

**Table 2:** Multivariate Cox regression models for progression-free and disease-specific survival

	Progression-free survival			Disease-specific survival		
Risk factor	HR	95% CI	*p*-value	HR	95% CI	*p*-value
T status T3–4 vs T1–2^1^	1.282	0.749–2.197	0.365	1.230	0.667–2.268	0.508
N status N+ vs N0^1^	1.655	0.804–3.409	0.171	**2.435**	**1.023–5.796**	**0.044**
Clinical staging IV vs I-III^1^	1.516	0.771–2.983	0.228	1.702	0.778–3.722	0.183
Tobacco current vs never/former^1^	**2.315**	**1.174–4.564**	**0.015**	1.732	0.876–3.426	0.114
HPV status^2^ Non-related vs related^1^	**0.412**	**0.200–0.849**	**0.016**	**0.356**	**0.157–0.805**	**0.013**
Subgroup GLO1^high/nuc^ vs GLO1^others, 1^	**1.784**	**1.054–3.018**	**0.031**	**1.791**	**1.037–3.094**	**0.037**

*HR* Hazard ratio*, CI* confidence interval*,* ^*1*^ *reference group,* ^*2*^ *related = viral DNA* ^*+*^ *RNA* ^*+*^ *, non-related = viral DNA* ^*+*^ *RNA* ^*−*^ *or viral DNA* ^*−*^ *according to* [20]

Significant results (*p* <0.05) (including the HR, 95% CI) are marked in boldface

Finally we addressed the question, whether the prognostic value of GLO1 staining patterns correlates with the mode of treatment and conducted subgroup analysis by univariate Cox regression analysis. While the prognostic value of nuclear GLO1 staining was largely independent of treatment with (*n* = 109) or without surgery (*n* = 46), a high GLO1 immunoreactivity score was significantly correlated with an unfavorable PFS after surgery (with or without adjuvant radio- or radiochemotherapy, Additional file 8: Fig. S3A). A similar trend was also found for DSS (Additional file 9: Fig. S3B). In contrast, a high GLO1 immunoreactivity score was significantly correlated with a poor clinical outcome in the absence of chemotherapy (*n* = 108), which was not observed for patients with chemotherapy (*n* = 47, Additional file 10: Fig. S4). All GLO1 staining patterns (high or nuclear GLO1 staining and their combination) were correlated with unfavorable PFS and DSS after radiotherapy (*n* = 131, adjuvant and definitive), but not for surgery only (*n* = 24, Additional Fig. S5). However, it is worth noting that the amount of cases without radiotherapy was limited in this retrospective cohort.

## Discussion

GLO1 is a central part of a ubiquitous detoxification system in the glycolytic pathway of normal and tumor cells, and enables cell proliferation and survival under dicarbonyl stress [4]. Accordingly, GLO1 overexpression was found in several human malignancies [3, 7], and is also a common feature in tumor tissue of primary OPSCC as demonstrated by immunohistochemistry in this study. High GLO1 expression was significantly correlated with the presence of AP modifications and administration of exogenous MG induced GLO1 protein levels in two HNSCC cell lines. Induced GLO1 expression as an adaptive response to elevated MG levels was reported previously for triple negative breast cancer cell lines [30]. These data support a compensatory induction of GLO1 expression as a common mechanism of tumor cells to counteract the cytotoxic effect of an MG accumulation, which appears to be a critical step in the pathogenesis and malignant progression of OPSCC. Accordingly, Cal27 with lower basal and MG-induced GLO1 expression were more sensitive in a colony-forming assay to the cytotoxic effect at high MG concentrations, which is consistent with the sensitization to exogenous MG of metastatic melanoma and prostate cancer cell lines after silencing of GLO1 expression [14, 31].

Patients with a high GLO1 expression had a significantly shorter progression-free and disease-specific survival, suggesting a critical role of GLO1 activity not only in the initiation and maintenance of malignant tumor growth, but also the invasive capacity and metastatic spread of OPSCC tumor cells. Indeed, a significant correlation between GLO1 overexpression and tumor cell invasion, lymph node metastasis as well as reduced 5-year survival was reported for gastric cancer [8]. Furthermore, a growing body of experimental evidence indicates a potential role for GLO1 in tumor cell motility and invasion, which was demonstrated by ectopic GLO1 overexpression or gene silencing in established tumor cell lines derived from gastric cancer [8], cutaneous SCC [13], and prostate cancer [15]. In this context it is also worth noting that high GLO1 expression has been linked to the activation of distinct key regulators in oncogenic signaling, such as NF-κB, AP1 and PI3K-AKT, which might contribute to tumor cell proliferation and survival, but also accelerated tumor cell motility, metastasis and treatment resistance [7].

In a substantial amount of OPSCCs with GLO1 expression, we detected a predominant nuclear staining in tumor cells. Nuclear GLO1 staining was already reported for human cutaneous basal cell carcinoma [13] and prostate cancer [15], and nuclear GLO1 translocation was shown in a cell culture model of murine fibrosarcoma [32]. But for the first time, we demonstrate that nuclear GLO1 translocation has a clinical impact as it is significantly correlated with shorter disease-specific survival. Furthermore, the combination of high and nuclear GLO1 staining serves as independent risk factor for an unfavorable outcome of OPSCC patients. So far, the mode of regulation and causal role of nuclear GLO1 in malignant progression and treatment failure remains largely elusive and will be a major challenge for future studies. It has been speculated that one consequence of DNA damaging therapy is a dramatic increase in MG formation due to active processes of DNA repair [3]. As a consequence DNA and nuclear proteins become modified, which might potentiate the cytotoxic effect of anti-tumor treatment. The presence of nuclear GLO1 might serve as a potent defense mechanism to protect key regulators of DNA repair and tumor cell survival in the nucleus from inactivation by MG-induced modification.

## Conclusions

In summary, the presented data support a critical role of GLO1 in the malignant progression and clinical outcome of OPSCC patients. Detection of a high and nuclear GLO1 staining pattern could be implemented in future clinical studies to identify OPSCC patients with a high risk for treatment failure, which might benefit from specific targeting of accelerated GLO1 expression and activity.

## Additional files

Additional file 1: **Table S1.** Summary of pathological and clinical data of the patient cohort (DOCX 29 kb)

Additional file 2: **Table S2.** List of primary and secondary antibodies (DOCX 44 kb)

Additional file 3: **Table S3.** Summary of the GLO1 immunoreactivity score, Arg-pyrimidine protein level and patient characteristics (XLSX 21 kb)

Additional file 4: **Table S4.** Summary of the GLO1 protein level and localization as well as patient characteristics (XLSX 25 kb)

Additional file 5: **Fig. S1.** Viability of FaDu and Cal27 cells at low concentrations of methylglyoxal. Assessment of the cytotoxic effect of MG at low concentration in a colony-forming assay. (TIFF 92 kb)

Additional file 6: **Fig. S2.** Impact of GLO1 inhibition on MG accumulation. Quantification of MG concentrations in cell culture supernations by HPLC. (TIFF 108 kb)

Additional file 7: **Table S5.** Univariate analysis of distinct risk factors for progression-free and disease-specific survival. (DOCX 72 kb)

Additional file 8: **Fig. S3.** Correlation of GLO1 staining patterns with PFS and DSS in patient subgroups stratified by surgery. Forrest plots for progression-free and disease-specific survival for subgroups of patients with or without surgery. (TIFF 178 kb)

Additional file 9: **Fig. S4.** Correlation of GLO1 staining patterns with PFS and DSS in patient subgroups stratified by chemotherapy. Forest plots for progression-free and disease-specific survival for subgroups of patients with or without chemotherapy. (TIFF 177 kb)

Additional file 10: **Fig. S5.** Correlation of GLO1 staining patterns with PFS and DSS in patient subgroups stratified by radiotherapy. Forest plots for progression-free and disease-specific survival for subgroups of patients with or without radiotherapy. (TIFF 178 kb)

## Abbreviations

AGEs: Advanced glycation end products
AP: Argpyrimidine
BrBzGSHCp_2_: S-*p*-bromobenzylglutathione cyclopentyl diester
DSS: Disease-specific survival
GLO1: Glyoxalase 1
HNSCC: Head and neck squamous cell carcinoma
HPV: Human papilloma virus
MG: Methylglyoxal
OPSCC: Oropharyngeal squamous cell carcinoma
PFS: Progression-free survival
