# Supplementary material for: Glyoxalase 1 expression is associated with an unfavorable prognosis of oropharyngeal squamous cell carcinoma
Source: BMC Cancer. 2017 May 26;17:382. doi: 10.1186/s12885-017-3367-5 (PMC5446730; doi:10.1186/s12885-017-3367-5)
Supplement: Supplementary file 1 — Table S1. Summary of pathological and clinical data of the patient cohort (DOCX 29 kb) [file 12885_2017_3367_MOESM1_ESM.docx]

**Additional Table S1. Summary of pathological and clinical data of the patient cohort**

| **Feature** | **Category** | **n** | **%** |
| --- | --- | --- | --- |
| Age [years] | 57.3 (38.4-87.1)^1^ | 156 | 100 |
| Gender | male | 116 | 74.4 |
|  | female | 40 | 25.6 |
| Tumor size | T1 | 17 | 11.0 |
|  | T2 | 53 | 34.2 |
|  | T3 | 30 | 19.4 |
|  | T4 | 55 | 35.5 |
|  | missing | 1 |  |
| Lymph node status | N0 | 31 | 20.0 |
|  | N1 | 24 | 15.5 |
|  | N2 | 92 | 59.4 |
|  | N3 | 8 | 5.2 |
|  | missing | 1 |  |
| Distant metastasis | M0 | 145 | 93.5 |
|  | M1 | 7 | 4.5 |
|  | Mx | 3 | 1.9 |
|  | missing | 1 |  |
| Clinical staging | I | 7 | 4.5 |
|  | II | 15 | 9.7 |
|  | III | 27 | 17.4 |
|  | IV | 106 | 68.4 |
|  | missing | 1 |  |
| Pathological grading | G1 | 8 | 5.7 |
|  | G2 | 68 | 48.6 |
|  | G3 | 55 | 39.3 |
|  | Gx | 9 | 6.4 |
|  | missing | 16 |  |
| Smoking history | never | 19 | 12.8 |
|  | former | 13 | 8.7 |
|  | current | 117 | 78.5 |
|  | missing | 7 |  |
| Alcohol consumption | never | 13 | 8.8 |
|  | former | 19 | 12.9 |
|  | current | 115 | 78.2 |
|  | missing | 9 |  |
| HPV status | non-related^2^ | 107 | 78.1 |
|  | related^3^ | 30 | 21.9 |
|  | missing | 19 |  |
| Primary therapy | S | 24 | 15.5 |
|  | RT | 21 | 13.5 |
|  | SRT | 62 | 40.0 |
|  | RCT | 25 | 16.1 |
|  | SRCT | 23 | 14.8 |
|  | missing | 1 |  |

*^1^ median age (range), ^2^ viral DNA-negative or DNA-positive but transcript-negative; ^3^ viral DNA- and transcript-positive according to (*[*Holzinger et al., 2012*](#_ENREF_1)*). S, surgery; RT, radiotherapy; RCT, radiochemo therapy.*
