# Supplementary material for: Glyoxalase 1 expression is associated with an unfavorable prognosis of oropharyngeal squamous cell carcinoma
Source: BMC Cancer. 2017 May 26;17:382. doi: 10.1186/s12885-017-3367-5 (PMC5446730; doi:10.1186/s12885-017-3367-5)
Supplement: Supplementary file 2 — Table S2. List of primary and secondary antibodies (DOCX 44 kb) [file 12885_2017_3367_MOESM2_ESM.docx]

**Additional Table S2. List of primary and secondary antibodies**

| Antigen | Cat. No. | Clone & Species | | Company | | Application | | Dilution | |
| --- | --- | --- | --- | --- | --- | --- | --- | --- | --- |
| Argpyrimidine | AGE06B | Monoclonal mouse | Biologo | | IHC | | 1:100 | |  |
| GLO1 | ab52835  ab81461 | Polyclonal mouse  6F10, monoclonal rat | Abcam  Abcam | | IHC  WB | | 1:100  1:1,000 | |  |
| β-Actin | A5441 | AC-15; monoclonal mouse | | Sigma-Aldrich | | WB | | 1:10,000 | |
| anti-rat-HRP | P0450 | Polyclonal rabbit | | Dako | | WB | | 1:10,000 | |
| anti-mouse-HRP | #7076 | Horse | | Cell Signaling | | WB | | 1:10,000 | |

*WB = Western blot, IHC = immunohistochemistry*
