# Supplementary figures and images for: Glyoxalase 1 expression is associated with an unfavorable prognosis of oropharyngeal squamous cell carcinoma
Source: BMC Cancer. 2017 May 26;17:382. doi: 10.1186/s12885-017-3367-5 (PMC5446730; doi:10.1186/s12885-017-3367-5)

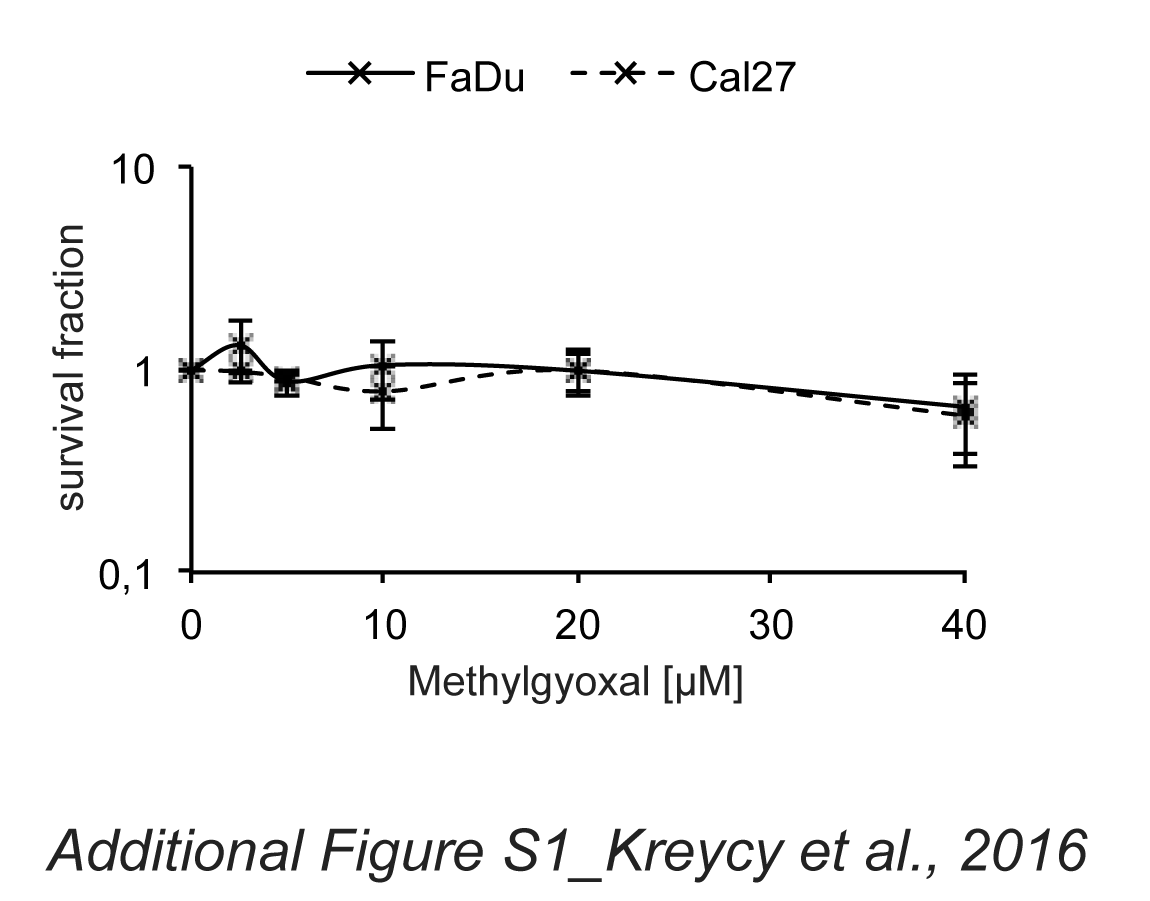

Supplement: Supplementary file 5 — Fig. S1. Viability of FaDu and Cal27 cells at low concentrations of methylglyoxal. Assessment of the cytotoxic effect of MG at low concentration in a colony-forming assay. (TIFF 92 kb) [file 12885_2017_3367_MOESM5_ESM.tif]

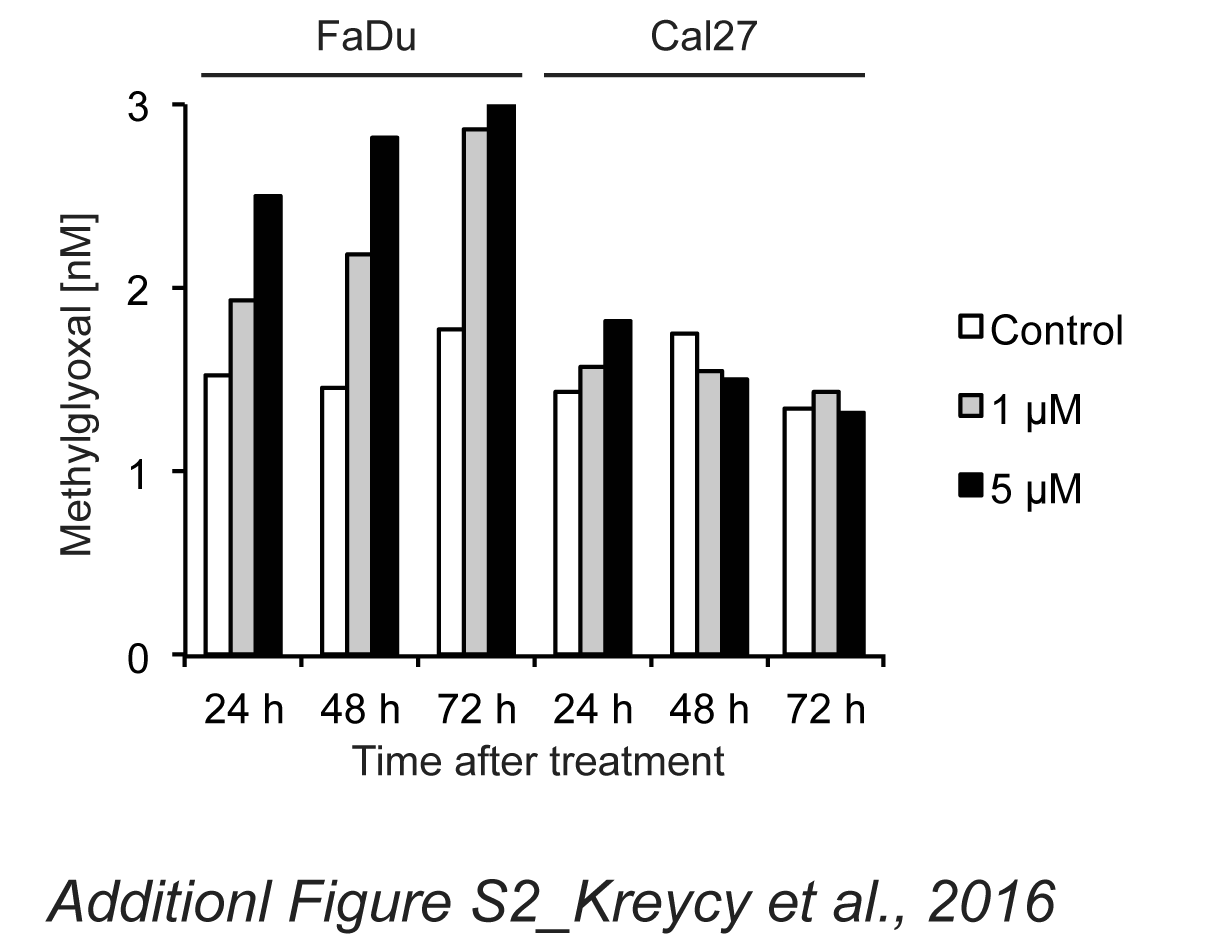

Supplement: Supplementary file 6 — Fig. S2. Impact of GLO1 inhibition on MG accumulation. Quantification of MG concentrations in cell culture supernations by HPLC. (TIFF 108 kb) [file 12885_2017_3367_MOESM6_ESM.tif]

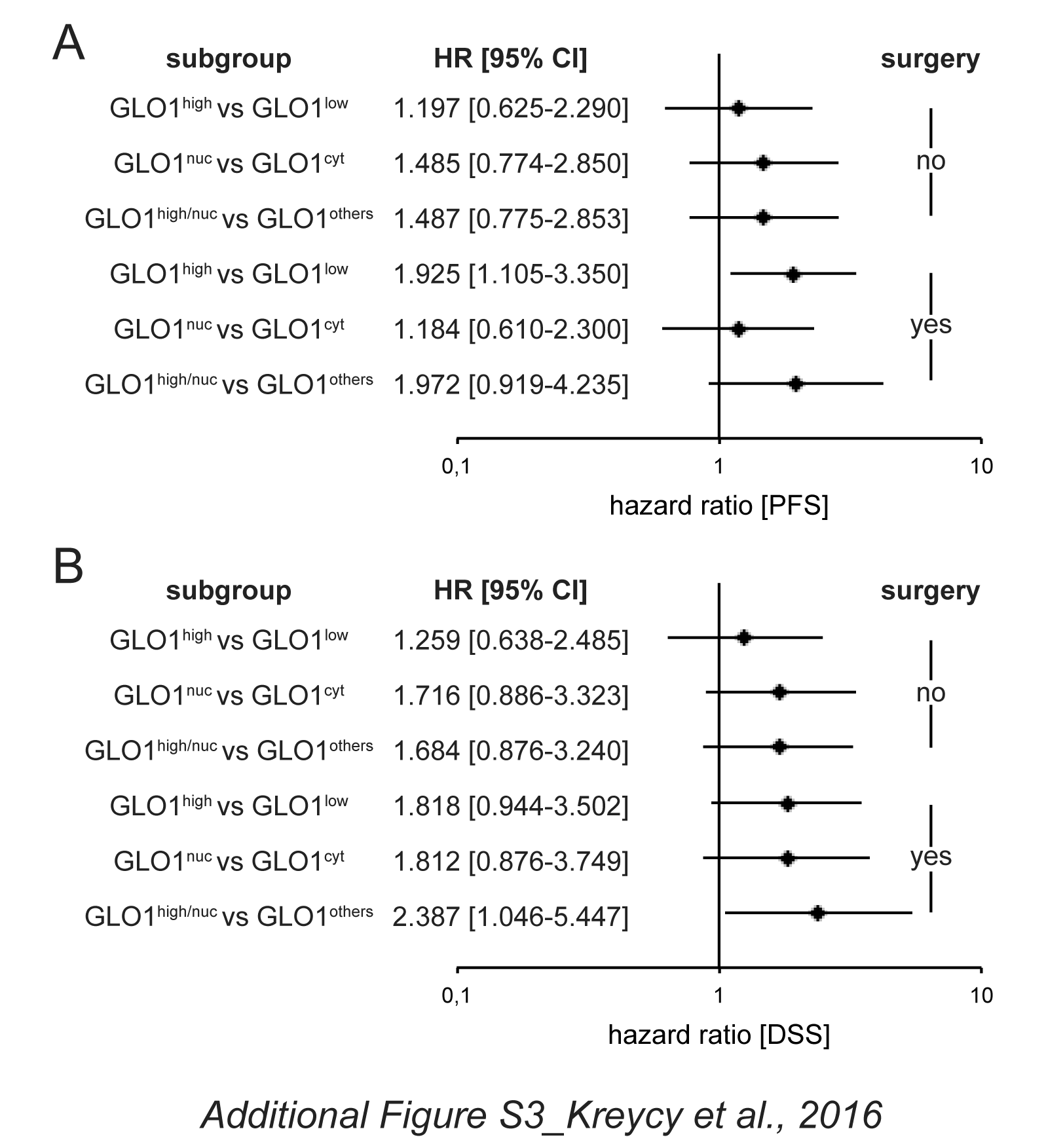

Supplement: Supplementary file 8 — Fig. S3. Correlation of GLO1 staining patterns with PFS and DSS in patient subgroups stratified by surgery. Forrest plots for progression-free and disease-specific survival for subgroups of patients with or without surgery. (TIFF 178 kb) [file 12885_2017_3367_MOESM8_ESM.tif]

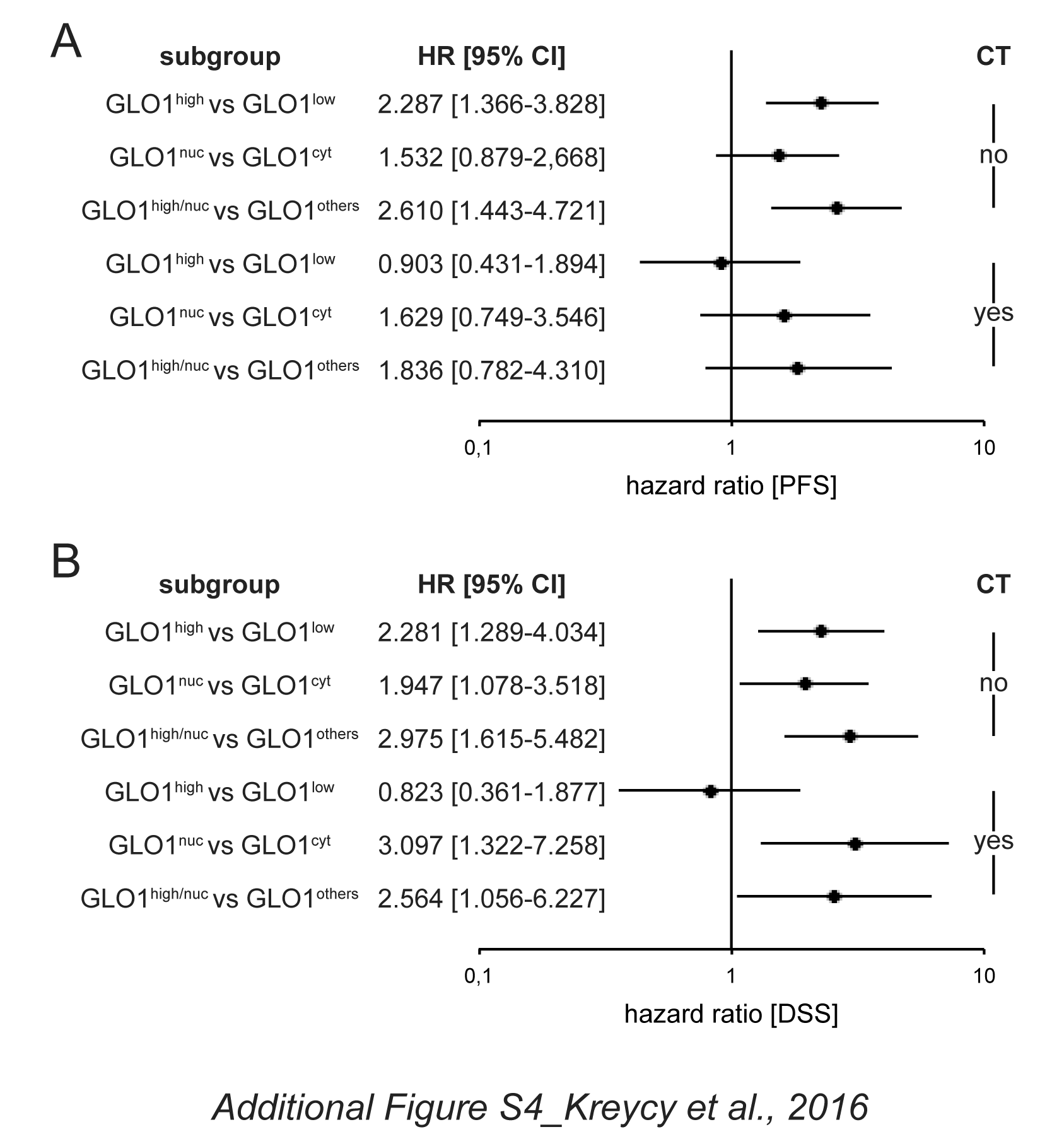

Supplement: Supplementary file 9 — Fig. S4. Correlation of GLO1 staining patterns with PFS and DSS in patient subgroups stratified by chemotherapy. Forest plots for progression-free and disease-specific survival for subgroups of patients with or without chemotherapy. (TIFF 177 kb) [file 12885_2017_3367_MOESM9_ESM.tif]

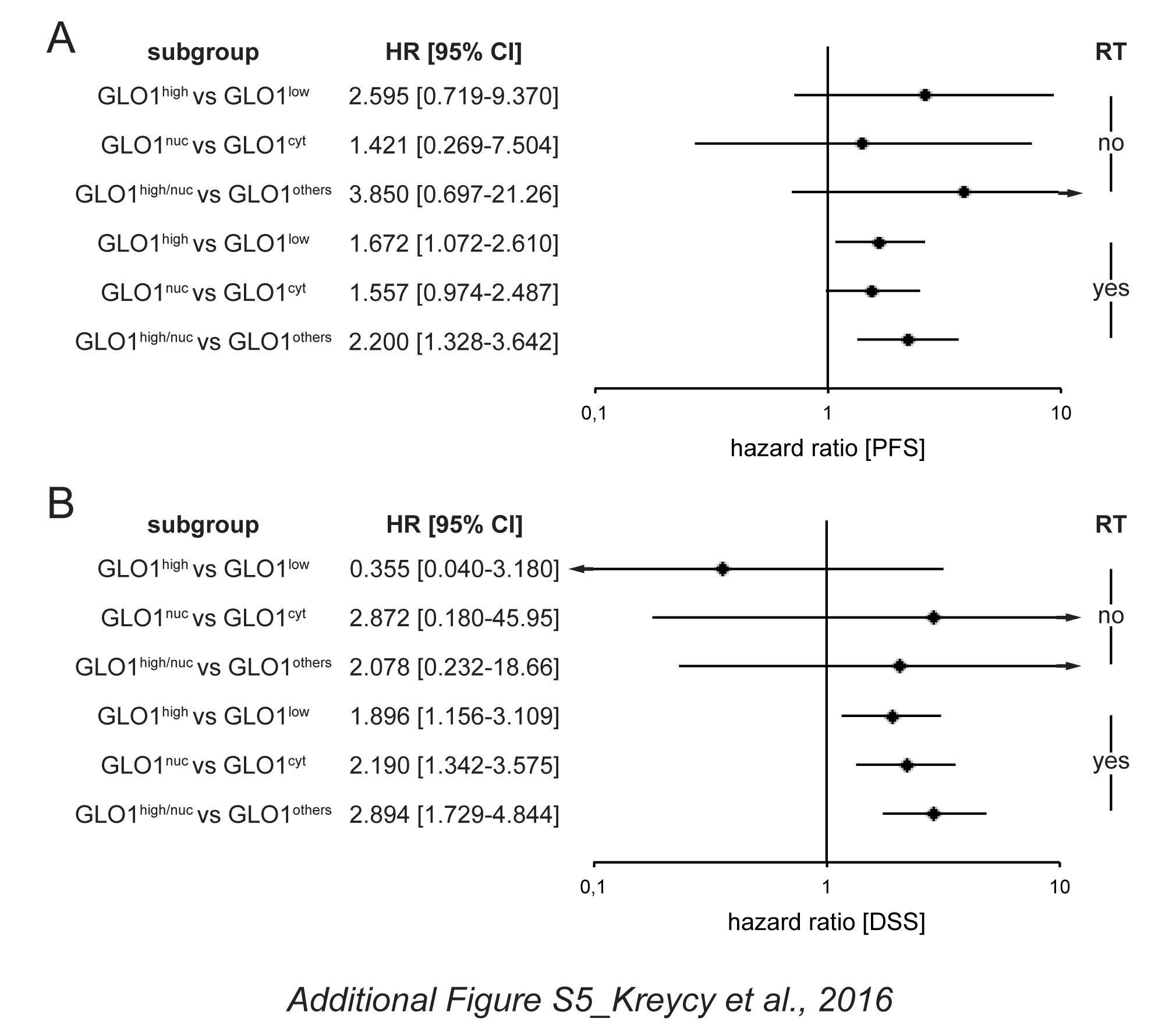

Supplement: Supplementary file 10 — Fig. S5. Correlation of GLO1 staining patterns with PFS and DSS in patient subgroups stratified by radiotherapy. Forest plots for progression-free and disease-specific survival for subgroups of patients with or without radiotherapy. (TIFF 178 kb) [file 12885_2017_3367_MOESM10_ESM.tif]
