# Supplementary material for: Glyoxalase 1 expression is associated with an unfavorable prognosis of oropharyngeal squamous cell carcinoma
Source: BMC Cancer. 2017 May 26;17:382. doi: 10.1186/s12885-017-3367-5 (PMC5446730; doi:10.1186/s12885-017-3367-5)
Supplement: Supplementary file 7 — Table S5. Univariate analysis of distinct risk factors for progression-free and disease-specific survival. (DOCX 72 kb) [file 12885_2017_3367_MOESM7_ESM.docx]

**Additional Table S5. Univariate analysis of distinct risk factors for progression-free and disease-specific survival**

|  | **Progression-free survival** | | | **Disease-specific survival** | | |
| --- | --- | --- | --- | --- | --- | --- |
| **Risk factor** | **HR** | **95% CI** | **p-value** | **HR** | **95% CI** | **p-value** |
| Gender  female vs male^1^ | 1.029 | 0.652-1.624 | 0.902 | 1.296 | 0.765-2.193 | 0.335 |
| Age [years]  ≥58.25 vs <58.25^1^ | 0.722 | 0.535-1.194 | 0.274 | 0.863 | 0.554-1.344 | 0.863 |
| T status  T3-4 vs T1-2^1^ | **2.112** | **1.382-3.228** | **0.001** | **2.419** | **1.479-3.957** | **<0.001** |
| N status  N+ vs N0^1^ | **1.883** | **1.083-3.272** | **0.025** | **3.144** | **1.510-6.545** | **0.002** |
| Pathological grading  G3 vs G1-2^1^ | 0.893 | 0.557-1.433 | 0.640 | 0.876 | 0.515-1.490 | 0.624 |
| Clinical staging  IV vs I-III^1^ | **1.825** | **1.155-2.883** | **0.010** | **2.680** | **1.524-4.713** | **0.001** |
| Alcohol  current vs never/former^1^ | 1.293 | 0.754-2.216 | 0.351 | 1.281 | 0.717-2.287 | 0.403 |
| Tobacco  current vs never/former^1^ | **2.505** | **1.365-4.595** | **0.003** | **1.951** | **1.031-3.693** | **0.040** |
| HPV status^2^  Non-related vs related^1^ | **0.341** | **0.171-0.680** | **0.002** | **0.318** | **0.146-0.693** | **0.004** |
| Subgroup  GLO1^high/nuc^ vs GLO1^others,1^ | **2.300** | **1.423-3.718** | **0.001** | **2.785** | **1.692-4.583** | **<0.001** |

*HR = Hazard ratio, CI = confidence interval, ^1^reference group, ^2^related = viral DNA^+^RNA^+^, non-related = viral DNA^+^RNA^-^ or viral DNA^-^ according to (*[*Holzinger et al., 2012*](#_ENREF_1)*).*
